# LLGL2 Increases Ca^2+^ Influx and Exerts Oncogenic Activities *via* PI3K/AKT Signaling Pathway in Hepatocellular Carcinoma

**DOI:** 10.3389/fonc.2021.683629

**Published:** 2021-06-10

**Authors:** Shusheng Leng, Fei Xie, Junyi Liu, Junyi Shen, Guangqian Quan, Tianfu Wen

**Affiliations:** ^1^ Department of Liver Surgery and Liver Transplantation Center, West China Hospital of Sichuan University, Chengdu, China; ^2^ General Surgery Department, Affiliated Hospital/Clinical Medical College of Chengdu University, Chengdu, China; ^3^ Department of Hepatobiliary, Pancreatic and Splenic Surgery, The First People’s Hospital of Neijiang City, Neijiang, China; ^4^ Central Laboratory, Affiliated Hospital/Clinical Medical College of Chengdu University, Chengdu, China

**Keywords:** LLGL2, Hepatocellular Carcinoma, PI3K/AKT signaling, Calcium influx, Progression

## Abstract

**Background:**

Lethal giant larvae (Lgl), scaffolding proteins, regulate the epithelial cell apicobasal polarity in Drosophila. They play important roles in asymmetric cell division, cell migration, and progenitor cells self-renewal as tumor suppressors. One of Lgl mammalian homologues proteins, LLGL2 overexpression has been reported in ER+ breast cancer and promotes tumor proliferation through regulating leucine uptake. Nonetheless, the role of LLGL2 in hepatocellular carcinoma (HCC) is still unknown.

**Methods:**

TCGA dataset mining, qRT-PCR, Western blot along with immunohistochemistry assays were employed to explore LLGL2 expression in human HCC samples and cell lines. Moreover, the clinical value of LLGL2 was investigated in 156 HCC patients. Furthermore, the role as well as the molecular mechanism of LLGL2 in the progression of HCC was explored through a series of *in vitro* and *in vivo* experiments.

**Results:**

LLGL2 was up-regulated in HCC tissues, which was related with certain clinicopathological features including tumor number, vascular invasion as well as advanced stage. High expression of LLGL2 predicted poor prognosis after hepatectomy. LLGL2 promoted HCC cells proliferation, migration and invasion through PI3K/ATK signaling by promoting calcium ion influx.

**Conclusion:**

Our study identified that LLGL2 is a tumor promoter in HCC for the first time, which could potentially be utilized as a new biomarker and a therapeutic target for HCC.

## Introduction

Hepatocellular carcinoma(HCC) is the 7^th^ leading most frequent cancer as well as the 3^rd^ leading cause of cancer mortalities globally in 2018, and about 841,080 new cases and 781,631 fatalities yearly (1). In China, HCC accounts for almost 50% of new cases and deaths annually (2). Despite the progress in operative techniques and targeted therapies, HCC prognosis has remained very poor. It is mainly because of high rates of relapse and metastasis after the treatments (3, 4). Understanding the pathogenesis and molecular mechanisms of HCC progression is essential for the development of novel and effective treatments for HCC.

Cell polarity is one of the most fundamental properties of normal cells (5, 6). It is essential for regulating multitude biological processes, including differentiation, proliferation, migration, adhesion, transformation and tumor formation (7, 8). Lethal giant larvae (Lgl), one of scaffolding proteins, which is a component of the epithelial apico-basal cell polarity machinery, controls the self-renewal and differentiation properties in progenitor cells acting as a tumor suppressor (9–12). The human homologues of Lgl are known as* *LLGL1 and LLGL2 (13). LLGL1 protein contains several conserved functional domains highly homologous to some regions of Lgl, which indicates that LLGL1 and Lgl proteins may have closely related functions (14, 15). Emerging evidences have shown that LLGL1 is a tumor suppressor in multiple human cancers, including hepatocellular carcinoma (12, 14, 16–20). LLGL2 is also associated with cancer progression. It has a vital function in the asymmetric cell division, cell migration and is essential for the development of placental labyrinth layer morphogenesis (21–23). Loss of LLGL2 in normal epithelium induced a mesenchymal phenotype (24). LLGL2 can suppress Snail-induced epithelial-mesenchymal transition (EMT), as a tumor suppressor preventing the dissemination of breast cancer (25). LLGL2 expression is either aberrantly localized or lost in gastric epithelial dysplasia and adenocarcinoma, as well as in pancreatic intraepithelial neoplasia and pancreatic ductal adenocarcinoma (26, 27). Apical membrane localization of LLGL2 was associated with lymphatic invasion and lymph node metastasis in lung adenocarcinoma (28). LLGL2 knockout causes epidermal cells tumorigenesis, which facilitate EMT by active ErbB signaling pathway (29). Nevertheless, LLGL2 does not always serve as a typical tumor-suppressor gene in mammalian (30). In human ER+ breast cancer, LLGL2 functions as a promoter of tumor growth, rather than a tumor repressor (31). Nonetheless, the biological as well as the clinical implications of LLGL2 in HCC are still unknown.

In our study, the expression of LLGL2 in HCC was investigated and its clinical significance for HCC prognosis was determined. Furthermore, LLGL2 was shown to enhance the intracellular calcium ion level, which in turn influences PI3K/AKT pathway signaling.

## Materials and Methods

### Bioinformatic Analysis

LLGL2 expression in HCC from TCGA database was performed by using online analysis tool GEPIA (32).

### Patient Specimens

Total 156 HCC samples were collected from Bio-bank of West China Hospital of Sichuan University, Affiliated Hospital/Clinical Medical College of Chengdu University and The First People’s Hospital of Neijiang between 2010 and 2017. All patients had not been subjected to any treatment before hepatectomy. All patients involved in our study were HBV (hepatitis B virus) associated HCC. Additionally, HCV (hepatitis C virus) or alcohol associated HCC were excluded. A total of 30 fresh HCC tissues and adjacent non-tumor liver tissue (ANLT) samples, which were acquired from West China Hospital of Sichuan University, were investigated using qRT-PCR. HCC histopathology was diagnosed by at least two independent pathologists. Paraffin-embedded tissues were employed to establish tissue microarray (TMA). All patients provided prior informed consent. Moreover, the Ethics Committee of West China Hospital of Sichuan University approved the study.

### Follow-Up and Prognostic Study

All subjects after hepatectomy were followed-up regularly by experienced researchers. The latest follow-up date was March, 2020. Alpha-fetpprotein (AFP) and ultrasonography were used to monitor HCC recurrence and metastasis every 3 month. When relapse was suspected, MRI (magnetic resonance imaging) and/or CT (computed tomography) scan were conducted. Overall survival (OS) along with Disease-free survival (DFS) was as the time from the date of hepatectomy to the date of mortality from HCC or to the date of local relapse or detection of distant metastasis. The detailed clinicopathological characteristics of these subjects are listed in detail in **Table 1**.

**Table 1:** Correlations between LLGL2 and clinicopathologic variables of HCC.

Clinicopathologic variable	No.	LLGL2		*P* value
		Low	High	
**Gender**				
Female	59	20	39	0.508
Male	97	38	59	
**Age (years)**				
≤50	90	28	62	0.260
>50	76	30	46	
**HBsAg**				
Positive	105	35	70	0.154
Negative	51	23	28	
**Liver cirrhosis**				
Presence	92	32	60	0.458
Absence	64	26	38	
**AFP**				
**<400μg/L**	87	33	54	0.827
**≥400μg/L**	69	25	44	
**Tumor number**				
Solitary	95	46	49	**<0.001**
Multiple (≥2)	61	12	49	
**Tumor size**				
≤5 cm	59	23	36	0.917
>5 cm	97	35	62	
**Vascular invasion**				
Presence	89	22	67	**<0.001**
Absence	67	36	31	
**Child-Pugh**				
**A**	97	32	65	0.165
**B**	59	26	33	
**Edmondson-Steiner grade**				
Low grade (I and II)	100	30	70	**0.013**
High grade (III and IV)	56	28	28	
**BCLC Stage**				
0/A	58	35	23	**<0.001**
B/C	98	23	75	

*Significant results (P < 0.05) are given in bold.

### Cell Lines and Cell Culture

Normal liver cell line L02 and human HCC cell lines including HepG2, Hep3B and Huh7 were supplied by the American Type Culture Collection (ATCC Rockville, MA). HCCLM3 and SMMC7721 were supplied by Shanghai Institute of Cell Biology, Liver Cancer Institute of Fudan University. We cultured the cell lines in DMEM added 10% fetal bovine serum, 1% penicillin as well as 1% streptomycin (Sijiqing, Hangzhou, China) at 37°C under 5% CO_2_.

### RNA Extraction and qRT-PCR

The TRIzol^®^ Reagent (Life Technologies, Carlsbad, CA) was employed to extract total RNAs from cells and frozen HCC tissues as outlined in the manufacturer protocol. cDNA was generated from the total RNA using the cDNA synthesis kit (ThermoFish Scientific, Shanghai, China). Thereafter, the SuperReal PreMix Plus kit (TIANGEN, Beijing, China) was employed to conduct the qRT-PCR following the protocol provided by the manufacturer. Sangon (Shanghai, China) synthesized the primers. GAPDH served as the internal control. The sequences of the primers are indicated in the **Supplementary Table S1**. The assays were replicated thrice.

### Western Blot Assessment

The RIPA lysis buffer (Solarbio, Beijing, China) was employed to extract proteins from the HCC cells as well as tissues. After that, the proteins were fractionated *via* SDS-PAGE. Then, the protein samples were transfer-embedded onto PVDF membranes (Millipore, Bedford, Mass). Afterwards, 5% skimmed milk was employed to block the membranes, followed by inoculation with the specified antibodies. Subsequently, enhanced chemiluminescence reagents (Thermo Scientific, Waltham, MA) were employed to explore the antigen-antibody complex. The primary antibodies: mouse LLGL2(1:500, Santa Cruz, CA, USA), Rabbit AKT (1:1000, Santa Cruz), Rabbit p-AKT(1:1000, Santa Cruz), Rabbit PI3K(1:2000, Santa Cruz) and Rabbit p-PI3K(1:2000, Santa Cruz) antibodies. mouse actin (1:3000, Sigma). HuaAn Biotechnology (Huaan, Hangzhou, China) supplied the corresponding secondary antibodies.

### Immunohistochemical Analysis

Human HCC tissue microarrays (TMA) slides consisted of 156 pairs of primary tumor tissues and ANLT specimens. The immunohistochemical-staining assay for LLGL2(1:50), Vimentin(1:100), E-cadherin(1:100) was conducted using a 2-step detection kit (Zhongshan Golden Bridge Biotechnology, Beijing, China).Antibody Vimentin and E-cadherin purchased from HuaAn Biotechnology (Huaan, Hangzhou, China). We evaluated LLGL2 expression with an inverted microscope (Olympus, Japan). The levels of LLGL2 expression were scored on the basis of the percentage of the positively stained tumor cells. The grading score were; 4 indicates >80% positive, 3 indicates 51-80% positive, 2 indicates 31-50% positive, 1 indicates 5-30% positive, and 0 indicates ≤5% positive. The level of LLGL2 protein expression was classified into a high expression group (2 - 4) and a low expression group (0 - 1) for downstream analyses (33).

### Vector Construction of LLGL2

GeneChem (Shanghai, China) supplied the LLGL2 knockdown lentivirus and the relative negative control (NC) lentivirus. To generate LLGL2 shRNA–resistant plasmids, synonymous mutations were introduced into the shRNA2 and shRNA3 which targeted region of WT LLGL2 cDNA clone, purchased from GeneChem (Shanghai, China) (pEGFP-N1-shRNAR-LLGL2).The sequences of the 3 candidate hairpin loops and pEGFP-N1-shRNAR-LLGL2 were indicated in the **Supplementary Table S2**. Transfection of the lentiviral harboring the short hairpin RNAs (shRNA) targeting LLGL2 into Hep3B as well as HCCLM3 cells was performed as per the protocol provided by the manufacturer. The Hep3B and HCCLM3 cells transfected with an empty vector served as the controls. The lentivirus transfection efficiency herein was >90%. The stably transfected lentivirus cell cells were utilized in the downstream experiments. Hep3B^sh2^ and HCCLM3^sh3^ cells were transiently transfected shRNA resistant-LLGL2 plasmid by using Lipofectamine 2000 (Invitrogen, Carlsbad, CA, USA) and analyzed 48h after transfection.Hep3B^NC^ and Hep3B^sh2^ cells were tested by genome sequencing (GeneChem, Shanghai, China).

### MTT and Colony Formation Assessments

To explore the cell proliferation level, we inoculated 5×10^3^ cells/well of Hep3B as well as HCCLM3 infected cells into 96-well plates. Six wells of every group were assessed daily. We treated the cells with 100μl fresh medium added MTT 0.5 mg/ml (Sigma, USA), followed by incubation at 37°C for 4 hours. After wards, the cells were suspended in 100μl of DMSO and subsequently shaken for 10 minutes at room temperature. The absorbance was determined at 570 nm. In the colony formation assessment, cells were planted into 35mm dishes (5×10^2^ cells/dish) and incubated at 37°C for 2 weeks. Crystal violet staining was performed to examine the colony formation.

For rescue assays, Hep3B^sh2^ and HCCLM3^sh3^ cells transiently transfected shRNA resistant-LLGL2 plasmid (Hep3B^sh2+LLGL2-shR^, HCCLM3^sh3+LLGL2-shR^). Cells were seeded as above. The proliferation capacity was detected by MTT and colony formation.

The intracellular Ca^2+^ chelator BAPTA/AM and PI3K inhibitor ADZ8186 was purchased from Selleckchem (Houston, TX, USA). Hep3B^NC^ and HCCLM3^NC^ cells were treated with BAPTA/AM (20 μM) or ADZ8186 (30μM), and then the proliferation capacity was detected by MTT and colony formation. Experiments were conducted in triplicate.

### Wound Healing and Transwell Assessment *In Vitro*

Wound Healing and Transwell invasion assessments were employed to determine the migration capacity of HCC cells knockdown LLGL2. In the Wound Healing assessment, Hep3B and HCCLM3 infected cells were inoculated into dishes and allowed to grow for 1 day. The cells grew to 90% confluence and then three scratch lines were made with a 10ul pipette tip. After wards, the rate of closure was explored after cultured for 24 or 48 hours. In the Transwell invasion analysis, we planted 1×10^5^ cells into the upper compartment of the insert with Matrigel coated membrane (BD Biosciences, Franklin Lakes, NJ), followed by incubation at 37°C for 24 hours. Thereafter, the cells as well as the gel in the upper compartment were carefully removed and staining of the cells which adhered to the underside of the membrane was performed using 0.1% crystal violet as well as 20% methanol. We counted the cell numbers using an inverted microscope (Olympus, Japan). For rescue assays, the migration capacity of Hep3B^sh2+LLGL2-shR^ and HCCLM3^sh3+LLGL2-shR^ cells was detected as above. The migration capacity of HCC cells treated with BAPTA/AM or ADZ8186 was evaluated. The assays were replicated thrice in each experimental group.

### Tumor Formation and Metastasis Assessment *In Vivo*

The orthotopic HCC mouse model was developed as described before (34). Briefly, Hep3B^sh2^, Hep3B^NC^ and HCCLM3^sh3^, HCCLM3^NC^ were administered subcutaneously into the left upper flank areas of the nude mouse (male, BALB/c, 3-4 weeks old). The size of the subcutaneous tumor was computed and recorded weekly using a Vernier caliper as indicated: tumor volume (mm^3^) = (L × W^2^)/2, (L: long axis; W: short axis), the measurements were repeated thrice. After 1 month, the subcutaneous tumor tissues were harvested and sectioned into commensurate segments of about 1 mm^3^. After that, we implanted each segment into the liver (n=5 mice/group). Following the elapse of 6 weeks after implantation, we sacrificed the mice, and the size for the tumors was computed at autopsy and volume (*V*) was computed *via* the formula: *V*=(L × W^2^)/2. Harvesting of the livers as well as the lungs from the sacrificed mice was done, followed by fixation using phosphate-buffered neutral formalin. Thereafter, histopathological analysis of the serial segments was performed *via* H&E (hematoxylin and eosin) staining, and the specialized pathologists recorded the metastatic foci. Subsequently, the tumor metastasis rate was compared between the groups of each panel. The mice were housed and manipulated as per the protocols which were approved by the Medical Experimental Animal Care Commission of West China Hospital of Sichuan University.

### Transcriptome Sequencing

RNA samples of Hep3B^NC^ and Hep3B^sh2^ cells were subjected to transcriptome sequencing. The transcriptome sequencing experiments were performed by GeneChem (Shanghai, China).

### Intracellular Calcium Measurements

Due to Hep3B and HCCLM3 cells were transfected with green fluorescence protein (GFP), the Rhodamine 2 indicator Rhod2 (YEASEN, Shanghai, China) was used to measure intracellular calcium. It emited an extensive fluorescence (λ = 578 nm) when it binded to the ion and after having been subjected to excitation λ = 549 nm. According to the manufacturer protocols, Hep3B^NC^, Hep3B^sh2^,HCCLM3^NC^ and HCCLM3^sh3^ cells were exposed to Rhod2 (5μM)for 30min at 37°C and protected from light. Then, a fluorescence microscope (Olympus BX 60 fluorescence microscope, Japan) was employed to acquire the images. And fluorescence intensity measured by Ultra Multifunctional Microplate Reader at 549nm and 578 nm. For rescue assays, the fluorescence intensity of Hep3B^sh2+LLGL2-shR^ and HCCLM3^sh3+LLGL2-shR^ cells was detected as above.

### Statistical Analyses

SPSS 21.0 software along with GraphPad Prism software were used to statistical analyses. Data were indicated as the mean ± SEM from at least three independent experiments. The Student’s t test was employed to assess the differences between two groups and variables data were assessed by Pearson χ^2^ or Fish exact test. The Kaplan-Meier approach was employed to generate the OS as well as DFS curves, and the log-rank test was employed to explore the survival differences between the high-LLGL2 expression group and low-LLGL2 expression group. Univariate along with the multivariate analyses were assessed using the Cox proportional hazards regression model to validate the independent risk factors. *P*< 0.05 signified statistical significance.

## Results

### LLGL2 Was Overexpression in HCC Cell Lines and HCC Tissues

**Figure 1:**
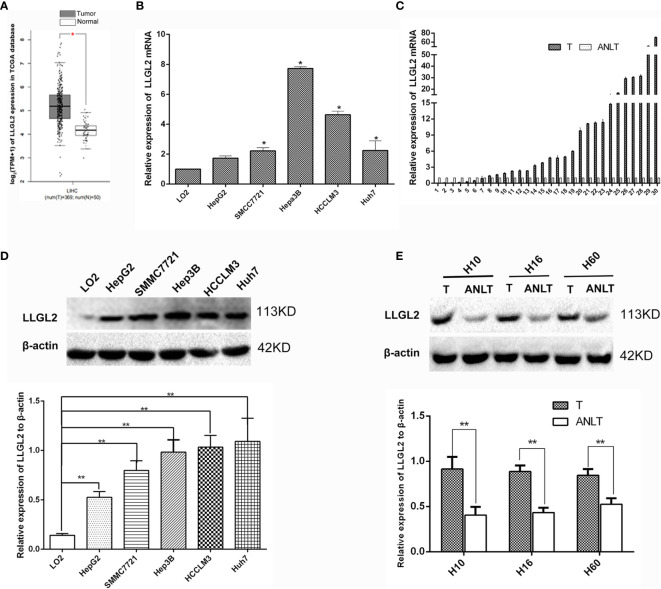
LLGL2 was highly expressed in HCC tissues and cells. **(A)** LLGL2 mRNA level of HCC patients from TCGA database were analyzed by using the GEPIA web tool. **(B)** The expression levels of LLGL2 mRNA in HCC cell lines were detected by quantitative real-time PCR (qRT-PCR). **(C)** LLGL2 mRNA expression levels were up-regulated compared with the adjacent nontumorous liver tissues (ANLTs) in 30 pairs of HCC tissues. Fold change was analyzed using the formula 2^-(ΔΔCT)^. **(D)** LLGL2 protein expression in HCC cell lines was detected by western blot. **(E)** LLGL2 protein expression in HCC tissues and ANLTs. GAPDH and β-actin was used as a loading control. * *P*<0.05; **P<0.01.

The GEPIA online analysis tool was utilized to explore the mRNA expression of LLGL2 in 369 HCC tissues as well as 50 normal liver tissues derived from the TCGA web data resource. The box plots demonstrated that mRNA expression of LLGL2 in HCC was overexpressed in contrast with their normal tissues (**Figure 1A**). To explore the expression of LLGL2 in HCC tissues and cells, qRT-PCR assays were conducted. Compared with L02 cells, which are immortalized human normal liver cells, LLGL2 mRNA was overexpressed in HCC cells (**Figure 1B**). To confirm the up-regulation of LLGL2, qRT-PCR was applied to analyze the LLGL2 expression in 30 pairs of HCC tissues and their matching ANLTs. LLGL2 expression was remarkably higher in the HCC tissues in contrast with that of ANLTs. Moreover, the median fold-change was 4.25 (**Figure 1C**). Furthermore, western blot was employed to analyze the levels of LLGL2 proteins in cell lines and tissues. Consistent with the results of qRT-PCR analysis, LLGL2 protein was overexpressed in HCC cells than in L02 cells (**Figure 1D**). It was also remarkably up-regulated in HCC tissues in contrast with ANLTs (**Figure 1E**).

### LLGL2 Was Related to Poor Clinicopathologic Features and Prognosis in HCC Patients

**Figure 2:**
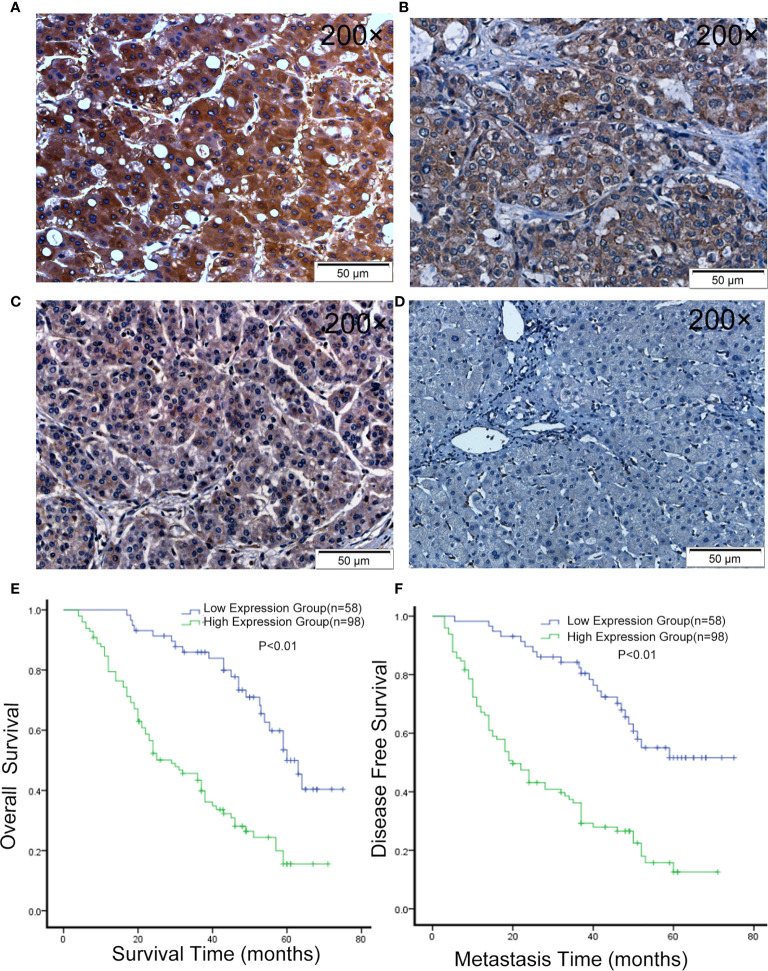
LLGL2 expression was up-regulated in HCC and was associated with poor prognosis. **(A–C)** Representative IHC images of LLGL2 expression in HCC tissues. **(D)** Representative IHC images of LLGL2 in ANLT. **(E)** The overall survival time of LLGL2 high expression group was significantly poorer than LLGL2 low expression group (*P*<0.01). **(F)** LLGL2 high expression group also had poorer disease-free survival than LLGL2 low expression group (*P*<0.01).

In order to further confirm this finding, we used 156 HCC samples from three centers to analyze LLGL2 expression levels by immunohistochemistry (IHC) assay. The findings demonstrated that LLGL2 was overexpressed in HCC tissues in contrast with the matched ANLTs (adjacent nontumorous liver tissues) and LLGL2 protein was located in the cytoplasmic (**Figures 2A–D**).Then, on the basis of the IHC results, the patients were cluster in to the high expression group (n=98) as well as the low expression group (n=58). We evaluated the relationship of LLGL2 expression with clinicopathologic characteristics of HCC patients. The data demonstrated that LLGL2 expression is linked to tumor number (*P*<0.001), vascular infiltration (*P*<0.001), Edmondson-Steiner grade (*P*=0.013), Barcelona Clinic Liver Cancer (BCLC) Stage (*P*<0.001) (**Table 1**). The univariate analysis and subsequent multivariate survival assessment *via* the Cox proportional hazards model identified tumor number (Hazard ratios (HR): 2.378 *P*=0.012), vascular infiltration (HR:3.218, *P*=0.029), and LLGL2 expression (HR: 3.018, *P*=0.025) as independent risk indicators of Overall Survival (OS) (**Table 2**). The tumor number (HR: 3.375, *P*=0.013), vascular infiltration (HR: 4.129, *P*=0.017) and LLGL2 expression (HR: 3.010; *P*=0.037) were validated as independent risk indicators for Disease Free Survival (DFS) (**Table 3**).

**Table 2:** Univariable and multivariable analysis of overall survival (OS) and LLGL2 by Cox proportional hazards regression Model.

Variables	No.	Univariable Analysis		Multivariable Analysis	
		HR (95% CI)	*P* Value	HR (95% CI)	*P* Value
**Gender**					
Female	59	1			
Male	97	1.927(0.880-4.231)	0.167		NA
**Age (years)**					
≤50	90	1			
>50	76	0.274 (0.169-1.510)	0.426 43085		NA
**HBsAg**					
Positive	105	1			
Negative	51	0.759(0.630-1.469)	0.182		NA
AFP					
<**400μg/L**	87	1			
** ≥400μg/L**	69	1.827(0.866-6.246)	0.529		NA
**Tumor number**					
Solitary	95	1		1	
Multiple (≥2)	61	2.135(1.615-4.032)	**0.015**	2.378 (1.720 - 5.467)	**0.021**
**Tumor size**					
≤5 cm	59	1			
>5 cm	97	1.924(0.724-6.210)	0.318		NA
**Vascular invasion**					
Absent	89	1		1	
Present	67	2.689(1.815-4.510)	**0.0322**	3.218 (1. 752-5.131)	**0.029**
**Liver cirrhosis**					
Absent	92	1		1	
Present	64	3.128(1.830-6.644)	**<0.001**	3.828(0.940-8.128)	0.059
**Child-Pugh**					
A	97	1			
B	59	1.827(1.341-6.283)	0.587		NA
**Edmondson-Steiner grade**					
Low grade (I and II)	100	1			
High grade (III and IV)	56	0.685(0.458-1.681)	0.269		
**BCLC Stage**					
0/A	58	1			
B/C	98	2.143(1.638-7.652)	0.079		NA
**LLGL2 expression**					
Low	58	1		1	
High	98	2.959(1.935-7.528)	**0.016**	3.018 (2.029 - 7.359)	**0.025**

*Significant results (P < 0.05) are given in bold. HR, hazard risk ratio; CI, confidence interval; NA, not applicable.

**Table 3:** Univariable and multivariable analysis of disease-free survival (DFS) and LLGL2 by Cox proportional hazards regression model.

Variables	No.	Univariable Analysis		Multivariable Analysis	
		HR (95% CI)	*P* Value	HR (95% CI)	*P* Value
**Gender**					
Female	59	1			
Male	97	1.615(0.850-3.946)	0.382		NA
**Age (years)**					
≤50	90	1			
>50	76	1.361 (0.682-1.936)	0.264		NA
**HBsAg**					
Positive	105	1			
Negative	51	0.459 (0.226-1.928)	0.116		NA
**AFP**					
<**400μg/L**	87	1			
** ≥400μg/L**	69	2.128(0.816-5.841)	0.182		NA
**Tumor number**					
Solitary	95	1		1	
multiple(≥2)	61	3.565(1.678-6.632)	**0.018**	3.375(1.812-6.871)	**0.013**
**Tumor size**					
≤5 cm	59	1			
>5 cm	97	2.150(0.814-4.180)	0.676		NA
**Liver cirrhosis**					
Absent	89	1			
Present	67	2.606(1.295-4.952)	**0.041**	2.201(0.828-5.180)	0.082
**Vascular invasion**					
Absent	92	1		1	
Present	64	4.281(1.839-7.966)	**0.008**	4.129(1.912-9.318)	**0.017**
**Child-Pugh**					
A	97	1			
B	59	2.195(0.910-6.546)	0.216		NA
**Edmondson-Steiner grade**					
Low grade (I and II)	100	1			
High grade (III and IV)	56	0.815(0.318-6.180)	0.510		NA
**BCLC Stage**					
0/A	58	1			
B/C	98	2.343(0.737-6.280)	0.229		NA
**LLGL2 expression**					
Low	58	1		1	
High	98	3.276(1.805-8.218)	**0.031**	3.010(1.732-8.579)	**0.037**

*Significant results (P < 0.05) are given in bold. HR, hazard risk ratio; CI, confidence interval; NA, not applicable.

Herein, the tumor relapse rate was 65.4%(102/156). Kaplan-Maier assessment demonstrated that OS rate was remarkably lower in HCC patients overexpressing LLGL2 relative to those with low expression of this protein (**Figure 2E**). DFS rates were similarly lower in the LLGL2 high expression group (**Figure 2F**). Meanwhile, the one-, three-, five-year OS rates in LLGL2 high-expression group was remarkably lower compared to the LLGL2 low-expression group (56% vs. 93%;28% vs. 74%;17% vs. 36%,respectively, *P*<0.01), and the 1, 3, 5 years DFS rates in the LLGL2 high-expression group was also lower in contrast with that of the low-expression group (47% vs. 90%; 26% vs. 62%; 10% vs. 45%, *P*<0.01).

### LLGL2 Promoted HCC Cell Proliferation, Invasion and Metastasis *In Vitro*

**Figure 3:**
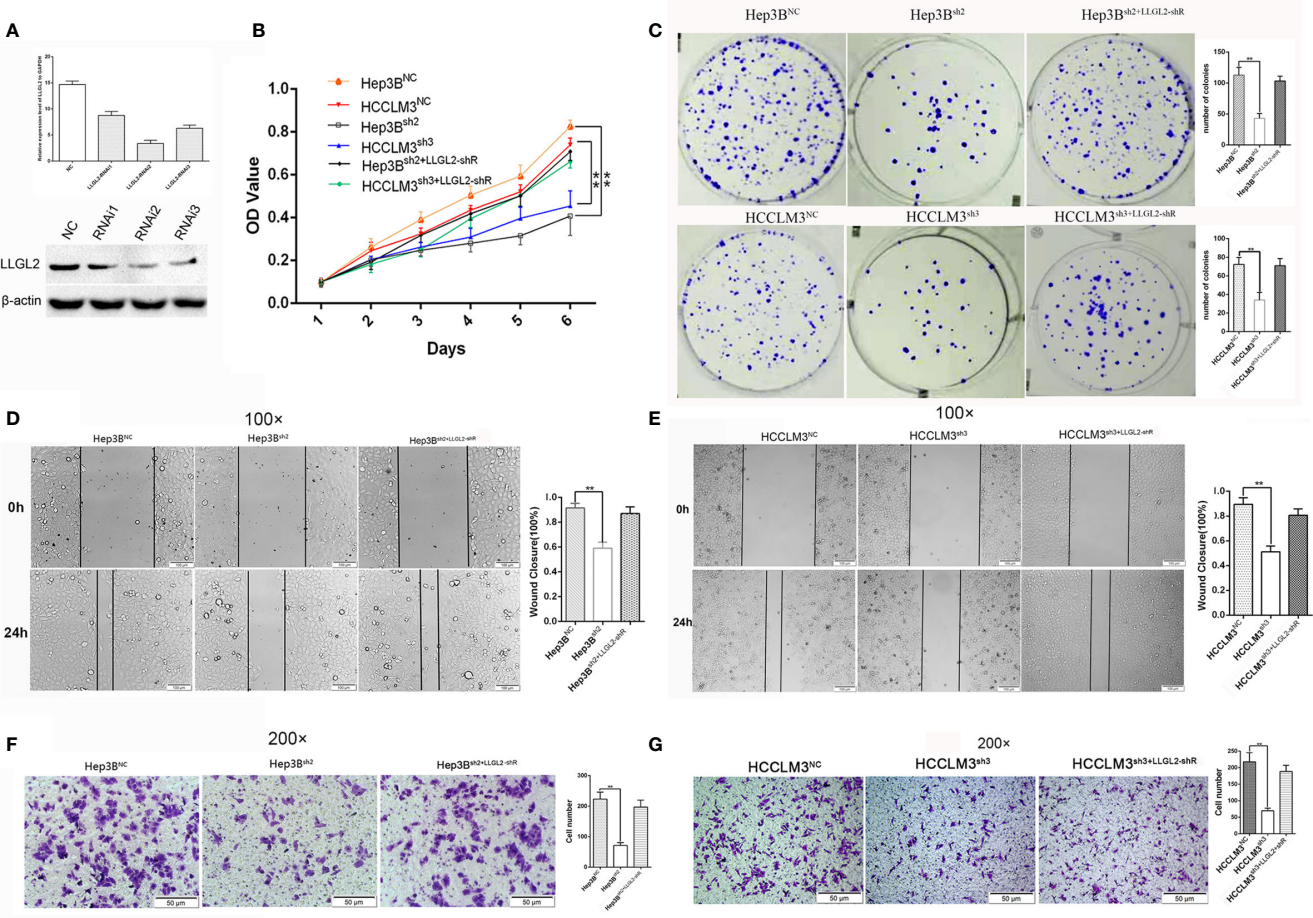
LLGL2 promoted proliferation and migration of HCC *in vitro*. HCC cells infected with or without LLGL2 knockdown lentivirus and then co-transfected with shRNA-resistant LLGL2 (LLGL2-shR). **(A)** The efficiency of LLGL2 knockdown in HCC Hep3B cells was assessed by qRT-PCR and western blot. LLGL2^sh2^ and LLGL2^sh3^ were selected for further study. **(B, C)**.The proliferation capacity of HCC cells were detected by MTT and colony formation assays The proliferation of Hep3B^sh2^ and HCCLM3^sh3^ were significantly weaker compared to control Re-expression of LLGL2-shR restored HCC cells proliferation capacity.**(D, E)** The closure of Hep3B^sh2^ and HCCLM3^sh3^ were significantly slower compared to control, and Re-expression of LLGL2-shR restored HCC cells migration capacity **(F, G)** The migration capacity of Hep3B^sh2^ and HCCLM3^sh3^ were weaker than control, and The migration capacity was restored after re-expression of LLGL2-shR. **P<0.01 based on the Students test. Error bars, standard deviation.

To further investigate the role of LLGL2 in the cell growth, invasion as well as metastasis *in vitro* and *in vivo*, Hep3B cells was infected with control (shRNA control) or LLGL2 silencing lentivirus (shRNA1,shRNA2,shRNA3). qRT-PCR along with the western blot analysis were employed to validate the knockdown efficiency. The results showed that the LLGL2-shRNA2 and LLGL2-shRNA3 could significantly reduce LLGL2 expression (**Figure 3A**). Then, we focused on characterizing the function on LLGL2 on proliferation as well as the migration of HCC cells.

The cell proliferation was evaluated *via* the MTT assay as well as colony formation assay. Compared to Hep3B^NC^ or HCCLM3^NC^ cells, LLGL2 knockdown decreased the proliferation of Hep3B or HCCLM3 cells (**Figure 3B**). Similar to the cell growth data, LLGL2 knockdown cells formed markedly fewer as well as smaller colonies in contrast with the control cells as indicated in **Figure 3C**. Then, we explored the influences of LLGL2 on cell migration by wound healing and transwell analyses. The results revealed that Hep3B^sh2^ or HCCLM3^sh3^ cells closed at a much slower rate and were less invasive relative to that of Hep3B^NC^ or HCCLM3^NC^ cells as indicated in **Figures 3D–G**. Next, we tested whether overexpression of shRNA-resistant LLGL2 restored proliferation and migration capacity in HCC/shLLGL2 cells by transfecting HCC/shLLGL2 cells with pEGFP-N1-shRNAR-LLGL2 or pEGFP-N1. The results showed that overexpression of shRNA-resistant LLGL2 restored HCC/shLLGL2 capacity of proliferation and migration (**Figures 3D–G**). These data implied that LLGL2 might facilitate the proliferation as well as the invasion potentialities of HCC cells.

### LLGL2 Promoted Tumorigenicity *In Vivo* Xenograft Tumor Model

**Figure 4:**
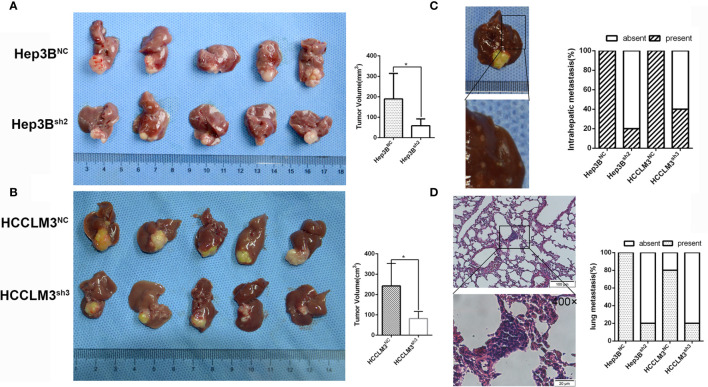
LLGL2 promoted HCC progression *in vivo*. The orthotopic HCC tumors transplantation model was established by using Hep3B^sh2^, HCCLM3^sh3^ cells and control cells. **(A)** The results showed that tumor volumes of Hep3B^sh2^ group were significantly smaller than that of Hep3B^NC^ group. **(B)** The results showed that tumor volumes of HCCLM3^sh3^ group were significantly smaller than that of HCCLM3^NC^ group. **(C, D)** Representative pictures of intrahepatic, lung metastasis; metastatic nodules proportion or lungs was calculated and compared. The rate of intrahepatic and lung metastasis in experiment groups were higher compared to control group. *P<0.05 based on the Students test. Error bars, standard deviation.

To further verify our results *in vitro*, we constructed models of orhtotopic xenograft tumors. Consequently, the tumors derived from the Hep3B^sh2^ cells were smaller, at the same time grew at a slower rate in contrast with the Hep3B^NC^ cell-derived tumors (**Figure 4A**). The tumors derived from the HCCLM3^sh3^ cells were also smaller and similarly grew at a slower rate in contrast with the control (**Figure 4B**). More significantly, in contrast with the control group, the intrahepatic and lung metastasis rates were decreased (**Figures 4C, D**). These data implied that LLGL2 could facilitate the metastatic potential of HCC *in vivo*.

### LLGL2 Was Linked to the PI3K/Akt Signaling Cascade by Enhancing Ca^2+^ Influx in HCC

**Figure 5:**
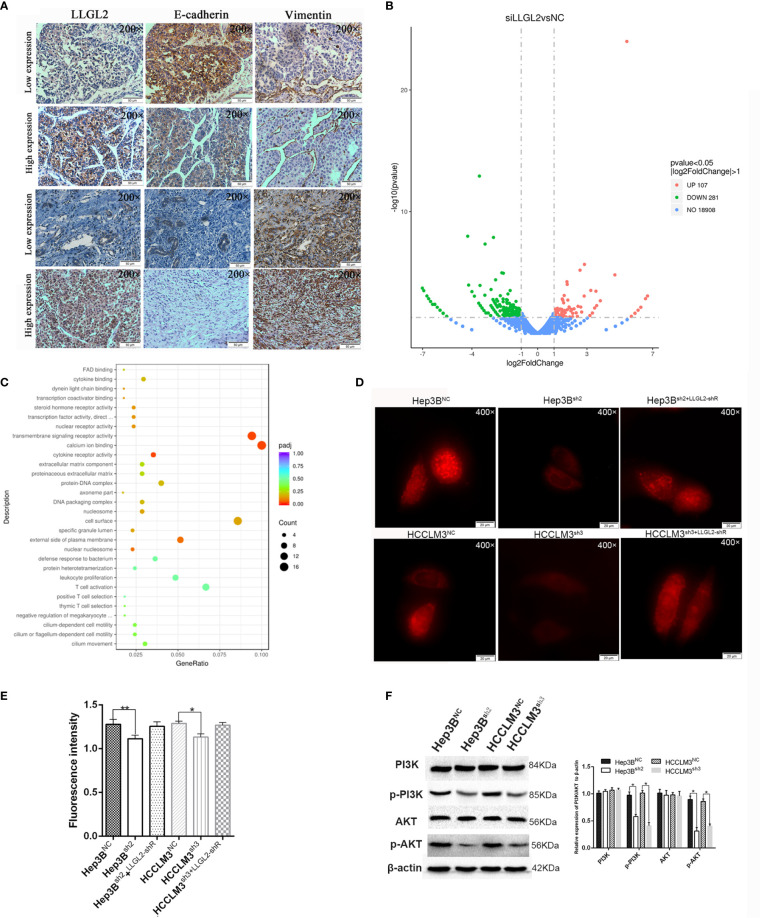
LLGL2 activated the Ca^2+^/PI3K/AKT signing pathway in HCC. **(A)** The expression of LLGL2 in HCC was not correlated with EMT in HCC tissues. LLGL2 and EMT biomarkers (E-cadherin, vimentin) were detected by IHC in HCC tissue microarrays. **(B)** Gene expression analysis of Hep3B cells in response to siRNA mediated depletion of LLGL2. **(C)** Transcriptome sequencing analysis for LLGL2 knockdown HCC cells. Relevant signaling pathways modulated by LLGL2 were assessed using Kyoto Encyclopedia of Genes and Genomes (KEGG) pathway enrichment analysis. **(D)** The concentration of Ca^2+^ in Hep3B and HCCLM3 cells measured with Rhod2,Images (×400) were captured on fluorescence microscope (Olympus BX 60 fluorescence microscope, Japan).**(E)** Quantification of Hep3B^NC^, HCCLM3^NC^ or Hep3B^sh2^, HCCLM3^sh3^ fluorescence intensity measured by Ultra Multifunctional Microplate Reader at 549 and 578 nm. **(F)** PI3K/AKT signaling pathway was suppressed by knockdown of LLGL2 through Western blotting. *P < 0.05. **P < 0.01.

**Figure 6:**
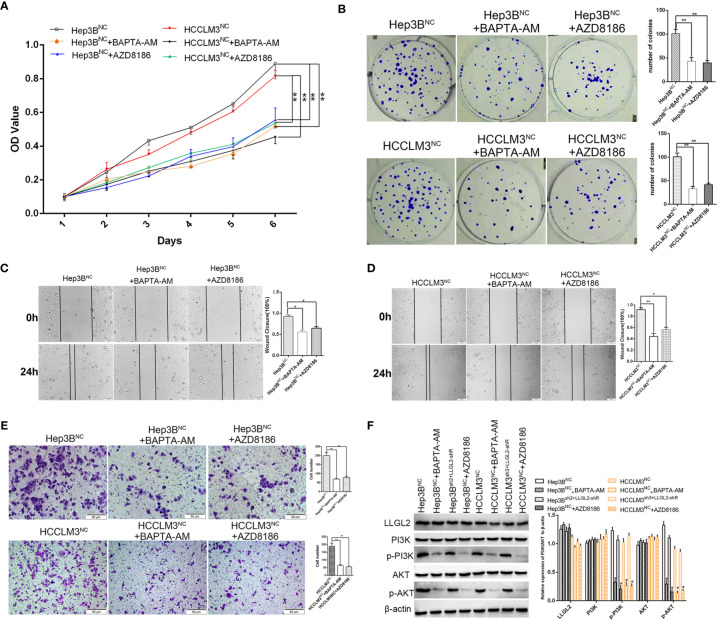
Molecular mechanism studies of LLGL2 roles in Ca^2+^/PI3K/AKT signing pathway combined inhibition. HCC cells of negative control treated with calcium chelators BAPTA-AM or with PI3K inhibitor AZD8186. **(A, B)**The proliferation capacity of HCC cells were detected by MTT and colony formation assays. The proliferation capacity of Hep3B^NC^ and HCCLM3^NC^ became weaker after treated with BAPTA-AM or AZD8186. **(C, D)** HCC cells treated with BAPTA-AM or AZD8186, the closure of cells were significantly slower compared to control. **(E)** The migration capacity of HCC cells treated with BAPTA-AM or AZD8186 were weaker than control. **(F)** HCC negative control cells co-transfected with shRNA-resistant LLGL2 (LLGL2-shR).The Western blotting results showed that LLGL2 activated Ca^2+^/PI3K/AKT signaling pathway. *P<0.05; **P<0.01 based on the Students test. Error bars, standard deviation.

Previous studies reported that LLGL2 participates in the epithelial-mesenchymal transition (EMT) (25).We analyzed the LLGL2 and EMT markers (Vimentin and E-cadherin) expression in the same human HCC tissue microarrays slides. The data demonstrated that LLGL2 expression was irrelevant with EMT in HCC (**Figure 5A**). To determine the molecular pathways associated with LLGL2-deficiency, we decided to investigate the gene expression profiles of Hep3B cells with LLGL2-deficiency through transcriptome sequencing (RNA-seq). Among the 19296 genes analyzed, 107 genes were up-regulated, whereas 281 genes was down-regulated (**Figure 5B**). The pathway analyses of these genes that were differentially expressed with the Kyoto Encyclopedia of Genes and Genomes (KEGG) point to the enrichment of the genes in the pathways associated with calcium ion binding, transmembrane signaling receptor activity (**Figure 5C**). Furthermore, PathCards data and literature reviews suggested that LLGL2 was involved in PI3K/AKT signaling pathway (35–37). Therefore, we speculated that LLGL2 might promote HCC progression through PI3K/AKT signaling cascade by enhancing Ca^2+^ influx in HCC. To confirm this hypothesis, we analyzed the Hep3B and HCCLM3 intracellular Ca^2+^ by using calcium indicator Rhod2-AM. Compared to the Hep3B or HCCLM3 control cells, the fluorescence intensity in the Hep3B^sh2^ and HCCLM3^sh3^cells were weaker (**Figure 5D**). After Rhod2 treatment, the fluorescence intensity of the Hep3B^sh2^ and HCCLM3^sh3^ cells was lower in contrast with that of the control (**Figure 5E**). Next, we detected fluorescence intensity of HCC/shLLGL2 cells which transiently transfected shRNA-resistant LLGL2.The results showed that the fluorescence intensity was restored in overexpression of shRNA-resistant LLGL2 HCC cells (**Figures 5D, E**).These results indicated that LLGL2 could increase Ca^2+^ influx in HCC cells. Then, we tested the AKT, p-AKT, PI3K and p-PI3K expressions *via* western blotting. The results demonstrated that p-PI3K and p-AKT expressions were significantly reduced in the LLGL2 knockdown Hep3B and HCCLM3 cells (**Figure 5F**). Furthermore, we treated HCC control cells with intracellular chelator BAPTA/AM or PI3K inhibitor ADZ8186. The results showed that the proliferation and migration ability of HCC cells decreased after treated with BAPTA/AM or ADZ8186 (**Figures 6A–E**). Overexpression of shRNA-resistant LLGL2 restored p-PI3K and p-AKT expressions *via* western blotting (**Figure 6F**). p-PI3K and p-AKT expressions were significantly reduced after treated with BAPTA/AM or ADZ8186 (**Figure 6F**). Taken together, these data showed that LLGL2 might be linked to the PI3K/Akt signaling axis by facilitating Ca^2+^ influx in HCC.

## Discussion

The results from this study suggest that LLGL2, the polarity gene, is a potential metastasis promoter of HCC, which may activate PI3K/Akt signaling by increasing intracellular calcium ions. We established for the first time that LLGL2 mRNA and protein were remarkably overexpressed in the HCC tissues as well as liver cancer cell lines. Overexpression of LLGL2 was linked to aggressive clinicopathological characteristics consisting of tumor number, vascular infiltration as well as advanced stage, and it was one of the independent risk indicators of OS and DFS in HCC. We also found that LLGL2 facilitated HCC cell proliferation, migration *in vitro*. In xenograft tumor model, LLGL2 acted as a tumor promoter in Hep3B grafts and promoted the lung dissemination of HCC cells. Notably, LLGL2 was not correlated with EMT. By performing transcriptome sequencing and PathCards analysis, we found that LLGL2 might be involved in Ca^2+^/PI3K/AKT signaling cascade. Re-expression of shRNA resistant LLGL2 rescued the proliferation and migration capacity of HCC/shLLGL2 cells, and reactivated Ca^2+^/PI3K/AKT signaling pathway. Therefore, our data indicated that LLGL2 might functioned as a tumor promoter in HCC through activating PI3K/AKT signaling cascade by enhancing Ca^2+^ influx.

Previous studies have concluded that LLGL2 is an apical-basal polarity scaffold protein, which performs diverse biological functions including cell division, migration, proliferation, and progenitor cells self-renewal in metazoans (9–12, 21, 38, 39). Mammalian LLGL2 is required for proper branching morphogenesis during placental development, but it does not function as a tumor suppressor gene (30). In the human breast cancer, LLGL2 serves as a promoter of tumor growth by aiding breast cancer cells to overcome nutrition stress (31). Consistent with this study, our results confirmed that LLGL2 was overexpressed in the human HCC and was linked to dismal prognosis of individuals with HCC. We also established that LLGL2 facilitated proliferation, migration as well as metastasis *in vitro* and *in vivo*. Thus, LLGL2 might be valuable for the prognosis along with targeted therapy of HCC.

Cell morphology and especially EMT is closely correlated with cancer progression and metastasis (34, 40, 41). Previous studies show that LLGL2 suppresses EMT *via* ErbB signaling, and pen/lgl2 mutant epidermal cells results in the development of epidermal tumors (29). LLGL2 upregulates epithelial proteins, E-cadherin and CK18, and suppresses Snail-induced EMT and metastasis (25). So we detected LLGL2 and EMT markers (Vimentin and E-cadherin) in the same human HCC tissue microarrays slides. Whereas we established that LLGL2 expression was not related to EMT in HCC. For further studying the molecular mechanism of LLGL2 promoting HCC progress, transcriptome sequencing analysis showed that LLGL2 might be involved in calcium ion signaling pathway. Intracellular calcium ions (Ca^2+^) as second messenger regulate gene transcription (42, 43). Accumulating evidences have indicated that Ca^2+^ signaling plays key roles in the tumor initiation, proliferation, migration, angiogenesis and metastasis (44–47). In this study, we found that LLGL2 could increase the concentration of intracellular Ca^2+^. The intracellular Ca^2+^chelator, BAPTA-AM, significantly blocked HCC cells proliferation and migration effects. These data indicated that LLGL2 promoted Ca^2+^ influx, and Ca^2+^ signal pathway might be a critical mechanism underlying LLGL2 promoted HCC progression. Intracellular calcium is associated with various signaling pathways (44). Previous studies show that intracellular calcium can promote HCC progression by activating PI3K/AKT pathway (35, 48). It is widely recognized that PI3K/AKT signaling cascade has a vital role in tumor initiation and progression (37, 49, 50). Through analyzing PathCards data, we found that LLGL2 is involved in PI3K/AKT pathway. The p-PI3K as well as p-AKT expressions were significantly reduced after LLGL2 knockdown in Hep3B cells. Furthermore, in this study, we used PI3Kβ inhibitor AZD8186 to treat HCC cells. We showed that AZD8186 inhibited PI3K-dependent activation of AKT and exerted as anti-tumor function in HCC (**Figure 6**).

Taken together, our study reveals that LLGL2 function as a tumor promoter by activating PI3K/AKT signaling through promoting Ca^2+^ influx in HCC. Further studies are needed to deeply explore this mechanism. Our study indicated that LLGL2 might be valuable prognostic marker as well as a promising therapeutic target in HCC.

## Data Availability Statement

The datasets presented in this study can be found in online repositories. The names of the repository/repositories and accession number(s) can be found in the article/**Supplementary Material**.

## Ethics Statement

The studies involving human participants were reviewed and approved by Ethics Committee of West China Hospital of Sichuan University. The patients/participants provided their written informed consent to participate in this study. The animal study was reviewed and approved by Ethics Committee of West China Hospital of Sichuan University.

## Author Contributions

Conception/design: SL, TW, and JL. Data collection and analysis: SL, TW, GQ, JS, and FX. Manuscript writing and polishing: SL, TW, and JS. All authors contributed to the article and approved the submitted version.

## Funding

This study was supported by grants from the State Key Scientific and Technological Research Programs (2017ZX10203207-003-0020), the Science and Technological Supports Project of Sichuan Province (2018SZ0204 and 2019YJ0149), the Health and Family Planning Commission of Sichuan Province (17PJ393), the Science and Technology Project of Chengdu (2018-YF05-01460-SN).

## Conflict of Interest

The authors declare that the research was conducted in the absence of any commercial or financial relationships that could be construed as a potential conflict of interest.
