# Supplementary material for: LLGL2 Increases Ca2+ Influx and Exerts Oncogenic Activities via PI3K/AKT Signaling Pathway in Hepatocellular Carcinoma
Source: Front Oncol. 2021 Jun 10;11:683629. doi: 10.3389/fonc.2021.683629 (PMC8223678; doi:10.3389/fonc.2021.683629)
Supplement: Supplementary file 1 [file Table_1.docx]

**Table S1.** List of the primer used in this study.

Name^a^  Sequence( 5’ 3’)

| LLGL2 | F | GCGTGTGTTCGAGATGGTGGAG |
| --- | --- | --- |
|  | R | TGGCTGCTGAGGAAGTGGTAGAG |
| GAPDH | F | AACGGATTTGGTCGTATTGG |
|  | R | TTGATTTTGGAGGGATCTCG |

^a^ F, forward primer; R, reverse primer.

**Table S2.** List of the three candidate interference sequences of LLGL2 used in this study.

| Sequences 1 | sense | 5′-ccggccCTTTCCTTGCAAAGCGATTctcgagAATCGCTTTGCAAGGAAAGggtttttg-3′ |
| --- | --- | --- |
| Sequences 2 | sense | 5′-ccggccAGTTTAACAAGACGGTGGActcgagTCCACCGTCTTGTTAAACTggtttttg-3′ |
| Sequences 3 | sense | 5′-ccggTGGCAACGTGTTTGTGGTGCActcgagTGCACCACAAACACGTTGCCAtttttg-3′ |
| LLGL2 NM_001015002(samesense) | sense | 5′-ATGAGGCGGTTCCTGAGGCCAGGGCATGACCCTGTGCGGGAGAGGCTCAAGCGGGACCTGTTTCAATTCAATAAAACTGTAGAGCATGGCTTCCCGCACCAGCCCAGCGCCCTCGGCTACAGCCCGTCCCTGCGCATCCTGGCCATCGGCACCCGTTCTGGAGCCATCAAGCTCTACGGAGCCCCAGGCGTGGAGTTCATGGGGCTGCACCAGGAGAACAACGCTGTGACGCAGATCCACCTCCTGCCCGGCCAGTGCCAGCTGGTCACCCTGCTGGATGACAACAGCCTGCACCTTTGGAGCCTGAAGGTCAAGGGCGGGGCATCGGAGCTGCAGGAGGATGAGAGCTTCACACTGCGTGGACCCCCAGGGGCTGCCCCCAGTGCCACACAGATCACCGTGGTCCTGCCACATTCCTCCTGCGAGCTGCTCTACCTGGGCACCGAGAGCGGTAATGTATTCGTAGTACAGCTGCCAGCTTTTCGTGCGCTGGAGGACCGGACCATCAGCTCGGACGCGGTGCTGCAGCGGTTGCCAGAGGAGGCCCGCCACCGGCGTGTGTTCGAGATGGTGGAGGCACTGCAGGAGCACCCTCGAGACCCCAACCAGATCCTGATCGGCTACAGCCGAGGCCTCGTTGTCATCTGGGACCTACAGGGCAGCCGCGTGCTCTACCACTTCCTCAGCAGCCAGCAACTGGAGAACATCTGGTGGCAGCGGGACGGCCGCCTGCTCGTCAGCTGTCACTCTGACGGCAGCTACTGCCAGTGGCCCGTGTCCAGCGAAGCCCAGCAACCAGAGCCCCTCCGCAGCCTCGTGCCTTACGGTCCCTTTCCTTGCAAAGCGATTACCAGAATCCTCTGGCTGACCACTAGGCAGGGGTTGCCCTTCACCATCTTCCAGGGTGGCATGCCACGGGCCAGCTACGGGGACCGCCACTGCATCTCAGTGATCCACGATGGCCAGCAGACGGCCTTCGACTTCACCTCCCGTGTCATCGGCTTCACTGTCCTCACAGAGGCAGACCCTGCAGCCAGTAGGAGAGCTTCGGGAGTGGGTGCCCAGGGTTAG-3′ |
